# CKS protein overexpression renders tumors susceptible to a chemotherapeutic strategy that protects normal tissues

**DOI:** 10.18632/oncotarget.22931

**Published:** 2017-12-04

**Authors:** John Tat, Céline Loriot, Martha Henze, Charles Spruck, Brunhilde H. Felding, Steven I. Reed

**Affiliations:** ^1^ Department of Molecular Medicine, The Scripps Research Institute, CA 92037, La Jolla, San Diego, USA; ^2^ Present address: Department of Medicine, University of California, San Diego, La Jolla, 92093-0652, California, San Diego, USA; ^3^ Tumor Initiation and Maintenance Program, Cancer Center, Sanford-Burnham-Prebys Medical Discovery Institute, La Jolla, 92037, CA, San Diego, USA

**Keywords:** cancer chemotherapy, cyclin-dependent kinase, cyclin-dependent kinase subunit, replication stress, replication stress checkpoint

## Abstract

The cyclin-dependent kinase-interacting proteins Cyclin-dependent Kinase Subunit 1 and 2 (CKS1 and 2) are frequently overexpressed in cancer and linked to increased aggressiveness and poor prognoses. We previously showed that CKS protein overexpression overrides the replication stress checkpoint activated by oncoproteins. Since CKS overexpression and oncoprotein activation/overexpression are often observed in the same tumors, we have hypothesized that CKS-mediated checkpoint override could enhance the ability of premalignant cells experiencing oncoprotein-induced replication stress to expand. This tumor advantage, however, could represent a vulnerability to exploit therapeutically. Here, we first show *in vitro* that CKS protein overexpression selectively sensitizes tumor-derived cell lines to nucleoside analog-mediated toxicity under replication stress conditions. A treatment combination of the nucleoside analog gemcitabine and an agent that induces replication stress (thymidine or methotrexate) resulted in selective targeting of CKS protein-overexpressing tumor-derived cells while protecting proliferative cells with low CKS protein levels from gemcitabine toxicity. We validated this strategy *in vivo* and observed that Cks2-overexpressing mammary tumors in nude mice were selectively sensitized to gemcitabine under conditions of methotrexate-induced replication stress. These results suggest that high CKS expression might be useful as a biomarker to identify subgroups of cancer patients who might benefit from the described therapeutic approach.

## INTRODUCTION

Cyclin-dependent Kinase Subunit (CKS) proteins are small (9 kDa) cyclin-dependent kinase (CDK)-interacting proteins expressed in all eukaryotes. Originally discovered in yeast [1–2], mammals possess two paralogs, Cks1 and Cks2, that share 81% amino acid homology [3]. The structure of the Cks1-Cdk2 complex has been solved crystallographically [4] and most research suggests that CKS proteins function as adaptors targeting cyclin-CDK complexes to CDK substrates [5], although additional functions have been reported. In mouse, germline deletion of both *CKS1* and *CKS2* results in early embryonic lethality, most likely because CKS proteins are essential for the expression of cyclins A and B and CDK1 [6]. Single gene deletions for *CKS1* [7] and *CKS2* [8] in mouse reveal specialized functions for the two paralogs in mammalian development. Cks1 is an essential accessory protein for the ubiquitin ligase SCF^Skp2^, mediating the ubiquitin-dependent proteolysis of the CDK inhibitors p27^Kip1^, p21^Kip1^, and the Rb-related protein p130 [7, 9, 10]. Consequently, *CKS1*^−/−^ mice are abnormally small. Cks2, the only paralog expressed in the germline, is essential for meiosis, as *CKS2*^−/−^ mice are sterile due to spermatocyte and oocyte arrest at meiotic metaphase I [8].

Of clinical interest, CKS proteins are frequently overexpressed in a broad spectrum of cancers, including those of the breast, cervix, colon, stomach, lung, and prostate [11–15], and overexpression is associated with poorer prognosis among breast cancer patients [16, 17]. Although a mechanistic link between CKS protein overexpression and oncogenesis is unclear, it may relate to the ability of CKS protein overexpression to override the replication stress checkpoint [18]. It is known that oncoprotein overexpression and/or activation can cause replication stress, which then activates the replication stress checkpoint to block subsequent DNA replication. This pathway limits tumor progression in the early stages of cancer development [19, 20]. We previously showed that in response to replication stress triggered by treatment with hydroxyurea or thymidine, or by overexpression of the oncoprotein cyclin E, CKS-overexpressing cells failed to arrest in S-phase [18]. They, instead, continued to fire replication origins, thus allowing DNA synthesis to persist. Therefore, CKS protein overexpression likely constitutes one mechanism by which premalignant cells with oncoprotein activation and/or overexpression can gain a proliferative advantage under conditions of replication stress, and develop towards an oncogenic phenotype.

While CKS overexpression-mediated checkpoint override may abet oncogenesis, it also reveals a potentially exploitable vulnerability for high-CKS protein-expressing tumors. Our previous findings [18] provide the rationale for the hypothesis being tested here: that cells with low CKS protein levels, when co-treated with an agent that promotes replication stress and a cytotoxic nucleoside analog drug, are protected from toxicity of the nucleoside analog due to activation of the replication stress checkpoint and resulting cessation of DNA replication. In contrast, tumor cells that overexpress CKS proteins continue to replicate DNA under conditions of replication stress, and are therefore selectively sensitized to nucleoside analog toxicity. We have tested this hypothesis in cell culture and *in vivo* using a breast cancer xenograft model. Our results show that treatment with a replication stress inducer is permissive for toxic nucleoside analog killing of CKS protein-overexpressing cells and tumors while protective for low CKS-expressing proliferative cells from nucleoside analog toxicity. This approach should therefore allow doses of nucleoside analog anti-cancer drugs that would otherwise not be tolerated.

## RESULTS

### Under conditions of replication stress, CKS protein overexpression correlates with poorer clonal survival after gemcitabine treatment

**Figure 1:**
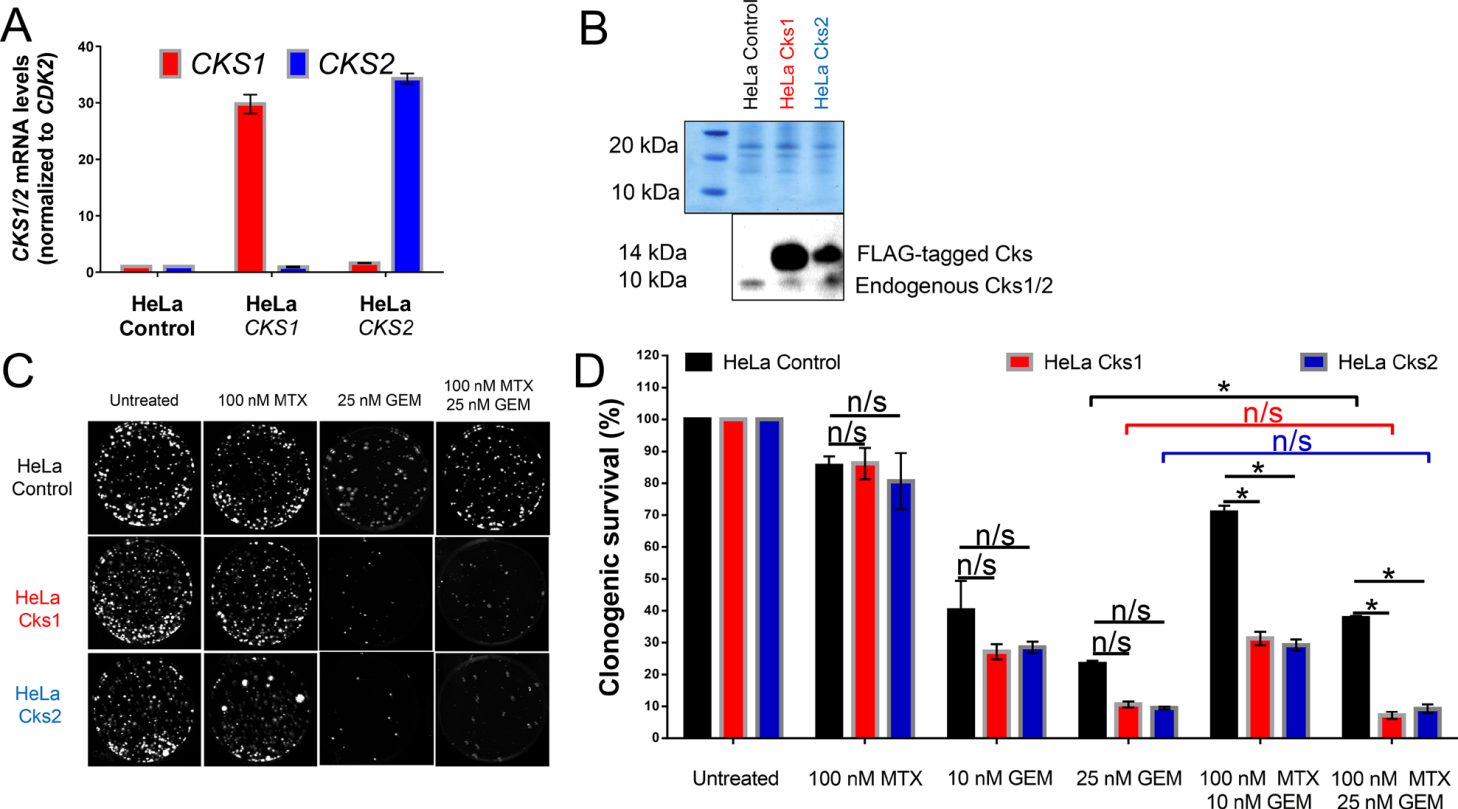
CKS protein overexpression sensitizes, while low CKS protein expression protects proliferating HeLa cells from gemcitabine-induced toxicity under replication stress qRT-PCR (**A**) and western blot (**B**) analyses of HeLa cells transduced with retrovirus to overexpress Cks1 or Cks2. *CKS* mRNA levels were normalized to *CDK2* mRNA. FLAG-tagged CKS protein levels were compared to controls with amido black staining used to calibrate loading. (**C**) Sample images of one representative clonogenic assay. (**D**) Quantification of two (*N* = 2) separate clonogenic assay experiments performed in triplicate (two-sided *t*-test: ^*^p < 0.05). Data are presented as normalized mean ± SEM.

To model tumors constitutively expressing high levels of CKS protein, HeLa cells were transduced with recombinant retroviruses to stably overexpress Cks1 or Cks2 (Figure 1A–1B). These HeLa transductants were then assessed for clonogenic survival in response to treatment with two drugs in clinical use: methotrexate (to induce replication stress), gemcitabine (a toxic nucleoside analog), or both combined. In CKS-overexpressing cells, methotrexate treatment had no effect on gemcitabine-mediated inhibition of colony formation, whereas controls with low CKS expression were protected from gemcitabine-mediated toxicity (^*^p = 0.04) (Figure 1C–1D). This finding supports the hypothesis that replication stress reduces the sensitivity of cells with low CKS protein levels to nucleoside analog toxicity.

Selective sensitization to gemcitabine under conditions of replication stress in cells expressing high levels of CKS protein was also established for HEK293A cells transduced to overexpress Cks1 or Cks2 (Supplementary Figure 1A–1B). Methotrexate protected control HEK293A cells with low CKS protein levels from gemcitabine-induced toxicity (^**^p = 0.004) (Supplementary Figure 1C).

**Figure 2:**
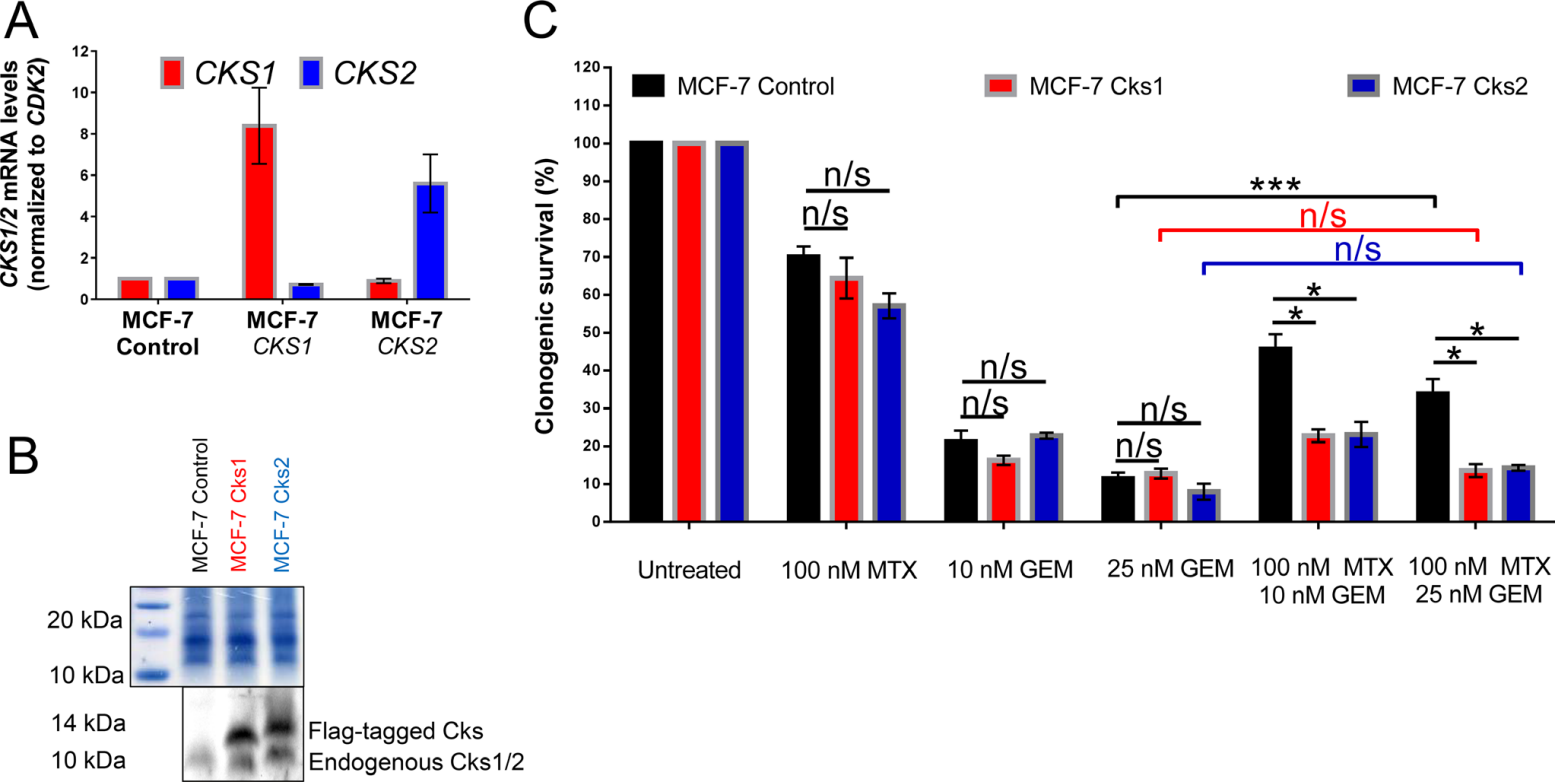
CKS protein overexpression sensitizes, while low CKS protein expression protects proliferating MCF-7 cells from gemcitabine induced toxicity under replication stress MCF-7, a mammary adenocarcinoma-derived cell line, was transduced with lentiviruses that overexpress Cks1 or Cks2 and expression was measured by qRT-PCR (**A**) and western blotting (**B**). *CKS* mRNA levels were normalized to C*DK2* mRNA levels. Amido black staining was used to calibrate loading. (**C**) Quantification of two (*N* = 2) separate clonogenic survival experiments performed in triplicate (two-sided *t*-test: ^*^p < 0.05 and ^***^p ≤ 0.001). Data are presented as normalized mean ± SEM.

Both models described above were carried out using cell lines that are not directly relevant to breast cancer, where CKS proteins are frequently overexpressed. The rationale for their use is that they are well-characterized, easy to culture, and grow rapidly. Therefore, to extend our findings in a breast cancer model, we stably overexpressed Cks1 or Cks2 in MCF-7 human breast cancer cells (Figure 2A–2B), which intrinsically express low levels of CKS proteins [22]. Methotrexate did not protect CKS-overexpressing MCF-7 cells, compared to low CKS controls, from gemcitabine-induced toxicity (^***^p = 0.001) (Figure 2C). Collectively, the results with HeLa, HEK293A, and MCF-7 cells support the hypothesis that CKS protein overexpression can sensitize tumor cells to a toxic nucleoside analog under conditions of replication stress.

### CKS overexpression sensitizes triple negative breast cancer cells to gemcitabine-induced toxicity under conditions of replication stress

**Figure 3:**
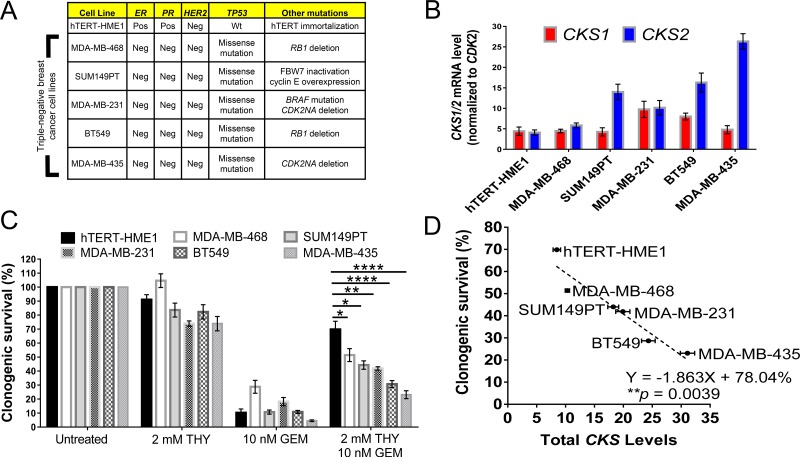
CKS expression levels in triple negative breast cancer cell lines correlate with gemcitabine sensitivity under replication stress (**A**) Overview of human triple negative breast cancer cell lines analyzed and their oncogenic mutations. hTERT-HME-1 cells are immortalized, non-transformed, human mammary epithelial cells used for comparison. Abbreviations: Pos = positive, Neg = negative, Wt = wild-type, ER = estrogen receptor, PR = progesterone receptor, HER2 = human epidermal growth factor receptor 2, *TP53* = tumor protein 53, *CDK2NA* = cyclin-dependent kinase inhibitor 2A, and *RB1* = retinoblastoma susceptibility protein. (**B**) Mammary epithelial and breast cancer cell lines stratified by *CKS1* and *CKS2* mRNA expression levels determined by qRT-PCR. *CKS1/2* levels were normalized to *CDK2* mRNA levels. *CKS1* and *CKS2* expression were estimated to be expressed approximately equal levels in HME-1 cells and mRNA levels were considered surrogates for protein levels. (**C**) CKS expression correlates with the level of protection from gemcitabine-induced toxicity under conditions of replication stress, based on clonogenic survival of mammary epithelial and breast cancer cells (two-sided *t*-tests: ^*^p < 0.05, ^**^p ≤ 0.01, and ^****^p ≤ 0.0001). Data are presented as normalized mean ± SEM. (**D**) Correlation between CKS protein expression and the level of protection from gemcitabine-induced toxicity under replication stress in human mammary and breast cancer cells, based on linear regression analysis (^**^p = 0.0039).

To further explore combining replication stress and gemcitabine treatment in cell-based models more relevant to breast cancer, we analyzed a panel of triple negative human breast cancer (TNBC) cell lines (Figure 2A). TNBC represents a subtype of breast cancer that lacks targeted therapies and is often treated with systemic anti-proliferative chemotherapy. For comparison, we included non-transformed but immortalized human mammary epithelial cells (HME-1) [23]. As antibodies that can distinguish Cks1 from Cks2 are not commercially available, we stratified these cell lines based on their intrinsic *CKS1* and *CKS2* mRNA expression levels and used mRNA expression as surrogates for CKS protein levels (Figure 3B). We then carried out clonogenic survival assays as described above, but in this case, used thymidine as the replication stressor to circumvent the possibility that tumors from which TNBC cell lines were derived may have acquired resistance to methotrexate. We observed that cell lines with low *CKS* mRNA levels were better protected from gemcitabine-induced toxicity than cell lines with high *CKS* mRNA levels (Figure 3C). Linear regression analysis showed that the level of *CKS* mRNA expression had a significant impact (^**^p = 0.0039) on the degree of replication stress-induced rescue from gemcitabine toxicity versus killing by this drug regardless of the type of disease driving mutations expressed (Figure 3A, 3D). We attempted to employ a complementary strategy of determining whether RNAi-mediated silencing of *CKS1* and *CKS2* in high-CKS-expressing TNBCs could restore replication stress-mediated rescue from gemcitabine. However, silencing of *CKS1* and *CKS2* led to extensive cell death (data not shown), suggesting that these lines are dependent on CKS overexpression. Nevertheless, it is noteworthy that HME-1 breast epithelial cells, which express the lowest levels of *CKS* mRNA among the analyzed cell types, exhibited the most robust thymidine-mediated rescue from gemcitabine toxicity. This result indicates that proliferating normal mammary epithelial cells can be protected from gemcitabine-induced toxicity when combined with a drug that induces replication stress.

### Under conditions of replication stress, CKS-overexpressing cells are killed more efficiently due to enhanced incorporation of gemcitabine into genomic DNA

**Figure 4:**
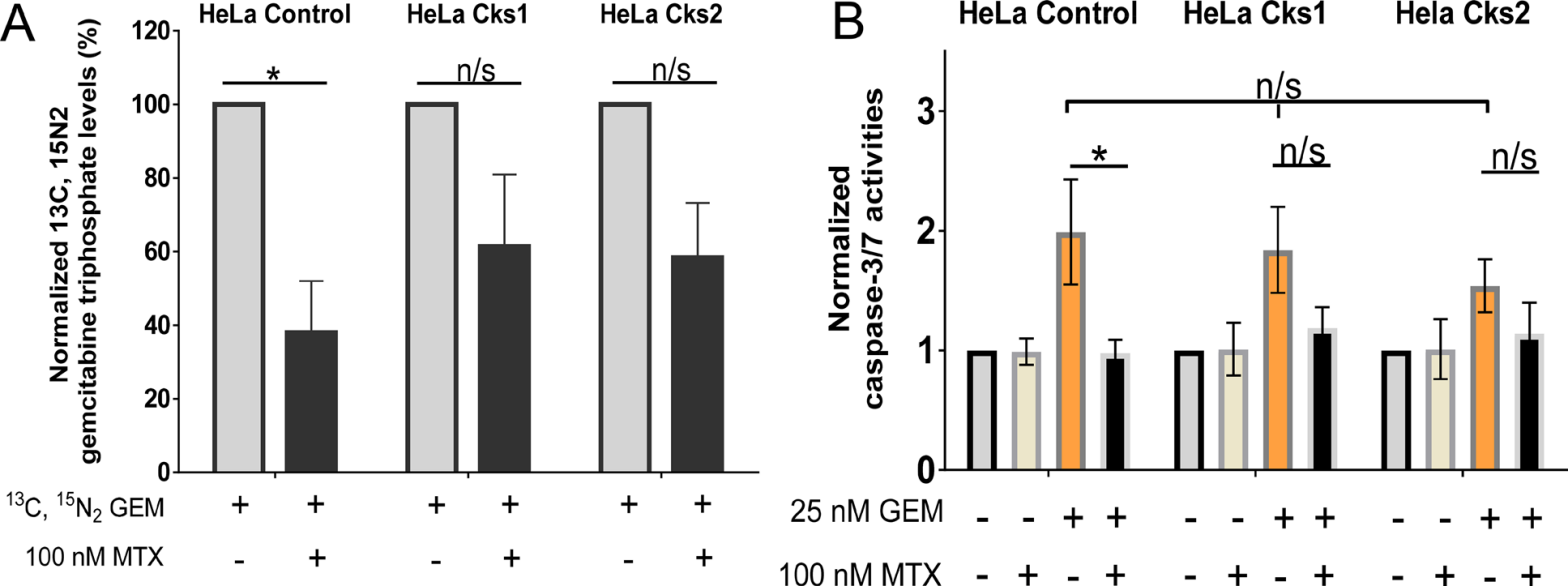
Increased gemcitabine incorporation into genomic DNA and enhanced apoptosis in high CKS-expressing cells under replication stress (**A**) Mass spectrometric analysis of gemcitabine incorporation into genomic DNA (*N* = 4 experiments) with or without replication stress (methotrexate treatment) (two-sided *t*-test: ^*^p < 0.05). (**B**) Induction of apoptosis based on caspase-3/−7 activation measured by fluorometric analysis (*N* = 4 experiments, each performed in duplicate) (two-sided *t*-test: ^*^p < 0.05). For both panels, data are presented as normalized mean ± SEM. Note that when treated with gemcitabine alone, all cell lines exhibited comparably high levels of apoptosis (one-way ANOVA: *p* = 0.75).

We sought to test whether the enhanced sensitivity of CKS protein-overexpressing cells to toxic nucleoside analogs under conditions of replication stress was due to continued DNA replication and nucleotide incorporation. Following cellular uptake, the pro-drug gemcitabine is phosphorylated by deoxycytidine kinase and other cellular kinases to its metabolically active forms, gemcitabine diphosphate (dFdCDP) and triphosphate (dFdCTP). The cytotoxic effect of gemcitabine is exerted in part by incorporation of dFdCTP into the elongating DNA strand [24]. Once a molecule of dFdCTP is added, only one additional nucleotide can be attached, resulting in irreparable chain termination and a subsequent load of DNA double-strand breaks, leading to apoptosis [24]. To evaluate how CKS protein overexpression affects dFdCTP incorporation into genomic DNA under conditions of replication stress, control and CKS-overexpressing HeLa cells were treated with heavy gemcitabine in the presence or absence of methotrexate for 48 hrs. Genomic DNA was then purified and subjected to multiple reaction monitoring mass spectrometry to quantify the amount of heavy dFdCTP incorporated. Under replication stress, heavy dFdCTP incorporation was significantly reduced in control cells with low CKS protein expression (62.1% reduction, ^*^p = 0.02), but not significantly in cells overexpressing either Cks1 (38.6% reduction, *p* = 0.14) or Cks2 (41.6% reduction, *p* = 0.07) (Figure 4A). Note that some reduction of dFdCTP incorporation is expected regardless of checkpoint function, since methotrexate, by reducing TTP levels, will slow replication fork progression.

The dFdCTP incorporation data suggest that reduced clonal survival of CKS-overexpressing cells in response nucleoside analog treatment is caused by increased cell death. To investigate this mechanism, HeLa cells expressing low versus high CKS protein levels were treated with methotrexate, gemcitabine, or a combination of the two drugs for 48 hrs, and then released from treatment for 48 hrs. Fluorometric assessment of caspase-3/−7 activation revealed that induction of replication stress significantly reduced apoptosis in gemcitabine-treated control cells with low CKS protein levels (50.9% reduction, ^*^p = 0.04). However, cells overexpressing Cks1 (35.2% reduction, *p* = 0.12) or Cks2 (26.45% reduction, *p* = 0.26) were not significantly protected from gemcitabine-mediated toxicity (Figure 4B). Thus, replication stress limits gemcitabine-induced apoptosis when CKS protein expression is low, but permits apoptosis-induced killing of cells with high CKS protein expression.

### CKS protein modulation of gemcitabine toxicity depends on S-phase entry

**Figure 5:**
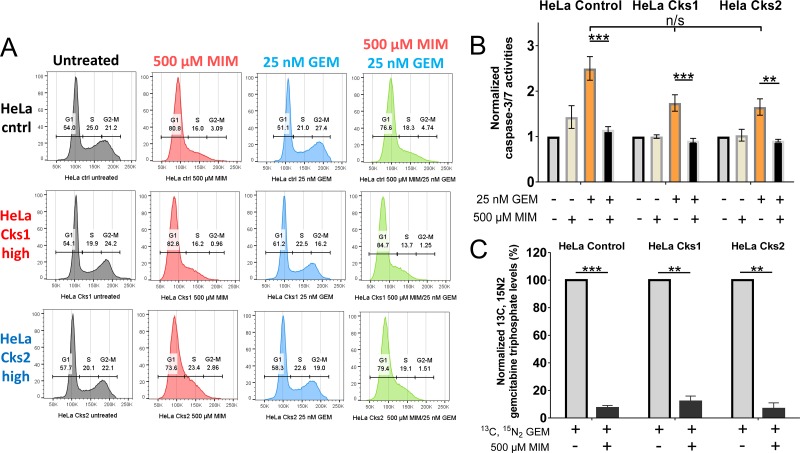
Entry into S-phase is required for gemcitabine incorporation and gemcitabine-induced apoptosis (**A**) Cell cycle distribution of HeLa cells after treatment with 500 μM mimosine (MIM) to arrest cells in G_1_, and/or 25 nM gemcitabine (GEM), analyzed by flow cytometry. X*-*axis represents DNA content, and Y-axis represents cell count. (**B**) Induction of apoptosis after treatment with mimosine and/or gemcitabine based on caspase-3/−7 activation measured by fluorometry (*N* = 2 experiments, each performed in duplicate) (two-sided *t*-test: ^**^p ≤ 0.01 and ^***^p ≤ 0.001). Note that cells treated with gemcitabine alone exhibited no significant difference in apoptosis, irrespective of CKS expression (one-way ANOVA: *p* = 0.6). (**C**) Quantification of gemcitabine incorporation into genomic DNA by mass spectrometric analysis (*N* = 3 experiments) (two-sided *t*-test: ^**^p ≤ 0.01 and ^***^p ≤ 0.001). Data in panels B and C are presented as normalized mean ± SEM.

Since nucleoside analogs are structurally similar to natural nucleosides that are incorporated into DNA, cancer drugs of this class are cell cycle-dependent and effective only against proliferating cells undergoing DNA replication. Thus, CKS protein-mediated sensitivity to nucleoside analogs should depend on S-phase entry. To test this hypothesis, HeLa cells with high versus low CKS protein expression were arrested in G_1_-phase of the cell cycle using mimosine, which induces expression of the CDK inhibitory protein p27^Kip1^ [25, 26]. The ability of gemcitabine to induce apoptosis was measured based on caspase-3/−7 activities (Figure 5). As expected, mimosine treatment for 48 hrs arrested most cells in G_1_-phase (Figure 5A). Importantly, G_1_-arrest affected gemcitabine-induced apoptosis regardless of CKS expression level (controls: 54.4% reduction, ^***^p = 0.0004; Cks1: 47.8% reduction, ^***^p = 0.001; Cks2: 45% reduction, ^**^p = 0.005) (Figure 5B). The reduction in apoptosis seen in G_1_-arrested cells was attributed to diminished incorporation of dFdCTP into cellular genomic DNA, as mimosine treatment caused significant reduction in heavy dFdCTP incorporation measured by mass spectrometry (controls: 92.8% reduction, ^***^p = 0.0004; Cks1: 88.1% reduction, ^**^p = 0.002; Cks2: 93.4% reduction, ^**^p = 0.002) (Figure 5C). Therefore, the observed enhancement of gemcitabine-induced toxicity in CKS-overexpressing cells depends on S-phase entry and continuing DNA replication.

### Increased incorporation of gemcitabine derivatives into DNA of CKS-overexpressing cells is caused by an override of the replication stress checkpoint

**Figure 6:**
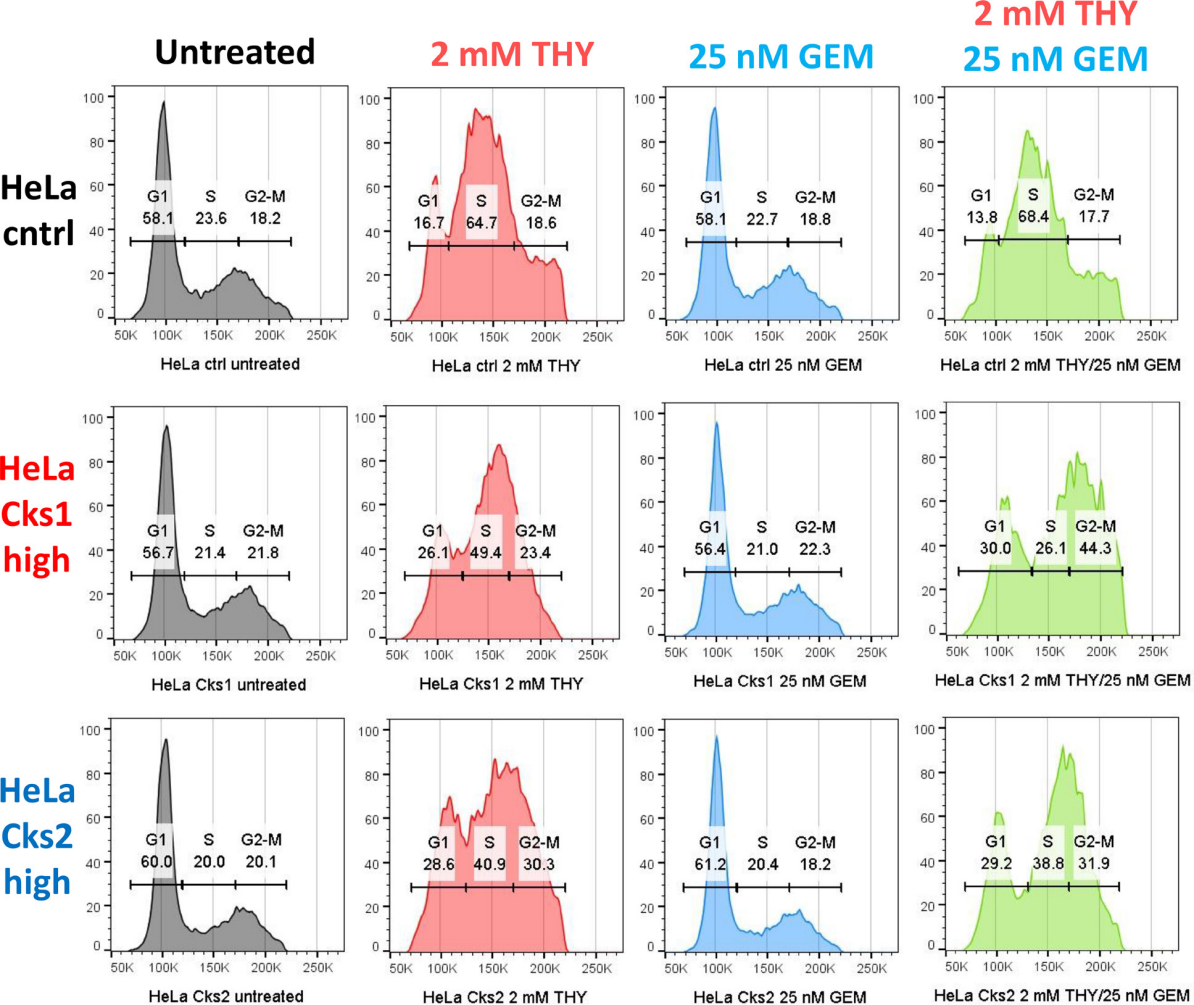
CKS-overexpressing cells continue to traverse S-phase despite replication stress Cell cycle distribution comparing HeLa cells with low CKS protein expression (ctrl) to their Cks1- and Cks2-overexpressing derivatives, treated with 2 mM thymidine (THY) and/or 25 nM gemcitabine (GEM) and analyzed by flow cytometry. X*-*axis represents DNA content, and Y-axis represents cell count.

We have reported that in response to replication stress, CKS-overexpressing cells fail to arrest in S-phase [18]. It is therefore likely that the increased incorporation of gemcitabine derivatives into the genomic DNA of CKS-overexpressing cells (Figure 4A), which leads to increased apoptosis (Figure 4B), can be attributed to an override of the replication stress checkpoint. To test this hypothesis, control and CKS-overexpressing HeLa cells were treated with thymidine, gemcitabine, or both for 48 hrs, and cell cycle distribution was analyzed by flow cytometry. Control cells largely arrested in S-phase (accumulation of cells between the 2N and 4N boundaries), whereas CKS-overexpressing cells continued to cycle, as evident by the proportion of cells in G_2_/M- and G_1_-phases (Figure 6).

### CKS protein overexpression sensitizes breast tumors to nucleoside analog toxicity under conditions of replication stress

**Figure 7:**
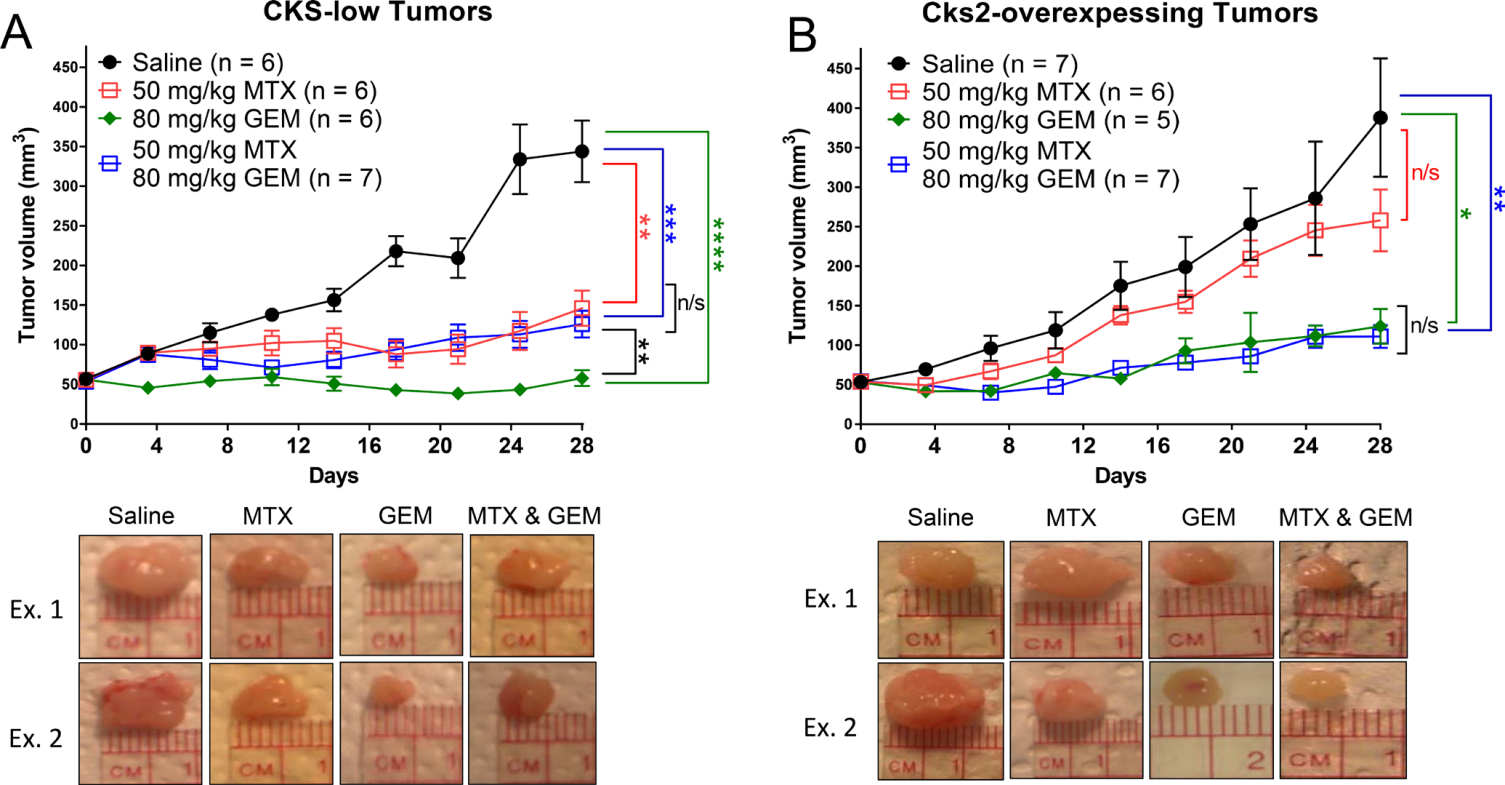
Cks2 overexpression sensitizes mammary tumors to gemcitabine induced toxicity under conditions of replication stress MCF-7 human breast cancer cells (1 × 10^6^) implanted into the fourth mammary fat pad of female nude mice were allowed to form tumors and reach ∼50 mm^3^ before biweekly treatments started. Graphs summarize changes in tumor volume over time and treatment responses to methotrexate (MTX), gemcitabine (GEM), or both drugs (two-sided *t*-tests: ^*^p < 0.05, ^**^p ≤ 0.01, ^***^p ≤ 0.001 and ^****^p ≤ 0.0001). Data are presented as mean ± SEM. (**A**) Low CKS protein expressing tumors and (**B**) Cks2-overexpressing tumors shown below each graph, “Ex.1” and “Ex. 2” represent randomly chosen example tumors (Day 28) from each treatment group.

In order to test the central hypothesis of the study in a more clinically relevant model, the therapeutic response of breast tumors expressing low versus high levels of Cks2 to combination treatment with methotrexate and gemcitabine was analyzed in an orthotopic mouse model. MCF-7 human breast cancer cells expressing intrinsically low levels of CKS proteins [22] and Cks2-overexpressing derivatives were implanted into the mammary fat pads of female nude mice. Tumors reached a size of ∼50 mm^3^ before the animals were treated biweekly with intraperitoneal injections of saline, methotrexate, gemcitabine, or methotrexate combined with gemcitabine. Tumor size was monitored for 28 days. Relative to saline controls, gemcitabine inhibited the growth of tumors bearing high or low levels of CKS proteins (CKS-low tumors: ^****^p < 0.0001; Cks2-overexpressing tumors: ^*^p = 0.02) (Figure 7A, 7B), consistent with the expected cytotoxicity of this nucleoside analog. When compared to saline controls, methotrexate significantly inhibited the growth of control tumors with low CKS protein levels (^**^p = 0.0014), but did not affect Cks2-overexpressing tumor growth (*p* = 0.37). This result is consistent with replication stress checkpoint-mediated arrest of tumors with low CKS protein levels, and an override of the replication stress checkpoint in Cks2-overexpressing tumors [18]. Notably, a combination of methotrexate and gemcitabine was less effective than gemcitabine alone in reducing the growth of low CKS-expressing tumors (^**^p = 0.006). In these tumors, the combination treatment effect was comparable to that seen with methotrexate alone (*p* = 0.45). This outcome suggests activation of the replication stress checkpoint by methotrexate can protect tumors with low CKS protein levels from gemcitabine-induced toxicity. Importantly, Cks2-overexpressing tumors in mice treated with methotrexate plus gemcitabine showed significant growth impairment compared to saline-treated control tumors (^**^p = 0.002). Growth inhibition of Cks2-overexpressing tumors with this combination treatment was comparable to that seen with gemcitabine alone (*p* = 0.78). This result suggests replication stress was unable to protect these tumors from nucleoside analog toxicity, consistent with our conclusions drawn from clonogenic cell survival assays (Figures 1, 2 and Supplementary Figure 1). Together, our findings support a model where Cks2-overexpressing tumors are sensitized to nucleoside analog toxicity under conditions of replication stress due to checkpoint override, whereas low CKS protein-expressing tumors are protected from gemcitabine by replication stress.

### Replication stress protects mice from nucleoside analog-induced systemic toxicity

**Figure 8:**
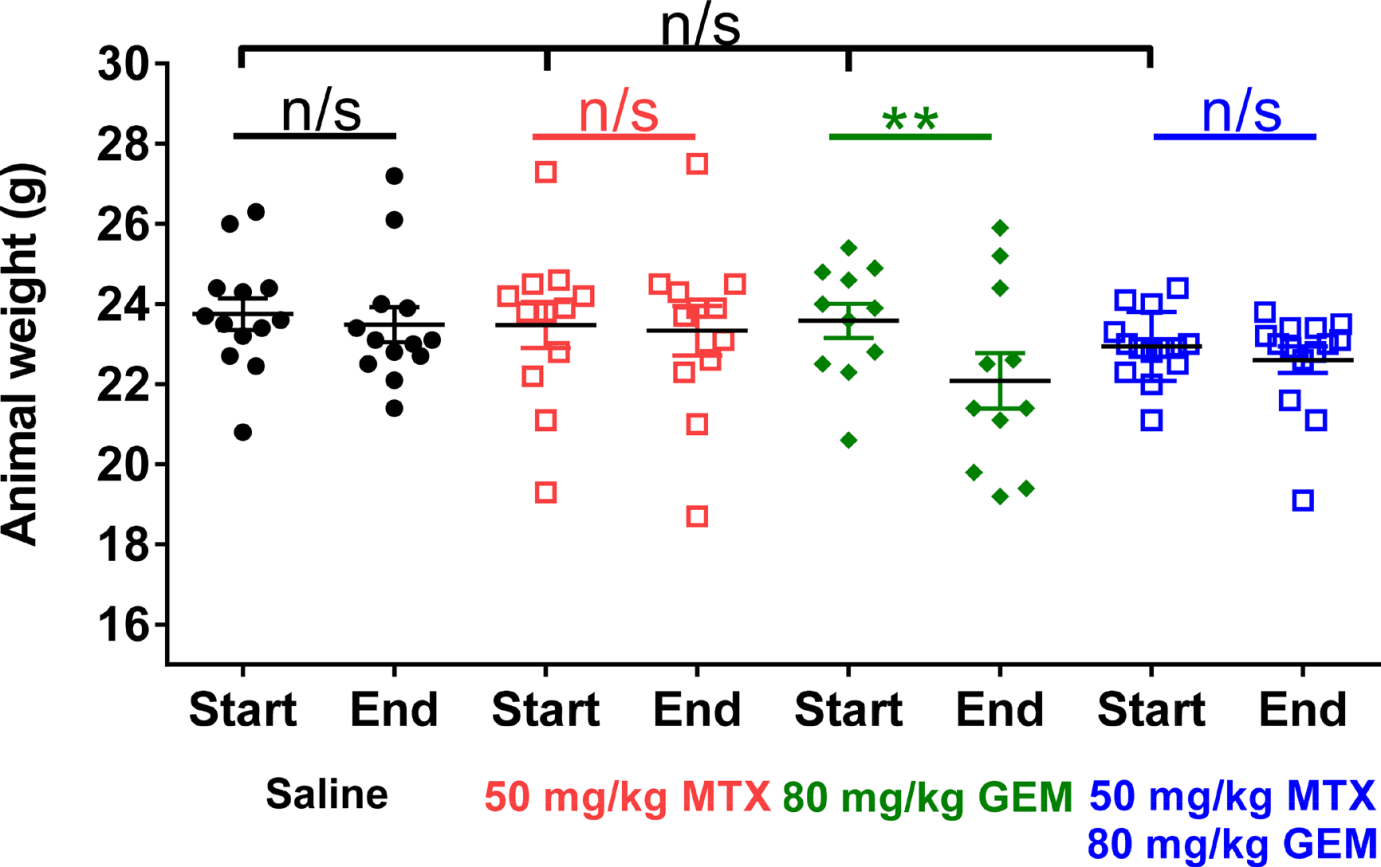
Methotrexate treatment prevents gemcitabine-induced weight loss in mice Whole body weights of all tumor-bearing mice in the study shown in Figure 7, before and after treatment with methotrexate (MTX), gemcitabine (GEM), or both drugs compared to saline control. At the beginning of treatment, mean body weight of all tumor-bearing mice was similar (one-way ANOVA: *p* = 0.5). Mean body weight for all tumor-bearing mice did not change over the course of treatment, except in the gemcitabine group (two-sided *t*-test: ^**^p = 0.01), as this treatment induced weight loss.

One limitation of chemotherapy is systemic toxicity, which non-specifically affects proliferating malignant and normal proliferating cells. Thus, therapeutic strategies are designed to selectively kill cancer cells while sparing proliferative healthy tissues. Our results show that under conditions of replication stress, CKS protein-overexpressing cells are more sensitive to gemcitabine-induced toxicity than cells with low CKS protein levels. Thus, replication stress, in principle, could be exploited to protect normal proliferating cells from gemcitabine-mediated toxicity *in vivo*. Indeed, the non-malignant immortalized HME-1 cell line provides support for this prediction. (Figure 3A). We found that this low CKS protein-expressing cell line (Figure 3B) was well-protected from gemcitabine-mediated toxicity by co-treatment with the replication stressor thymidine (Figure 3C–3D). In our animal model, we followed whole body weight during the course of treatment as a surrogate for systemic toxicity in each treatment group (Figure 8). We observed that mean body weight of tumor-bearing mice treated with saline or methotrexate alone remained unchanged throughout the study (*p* = 0.57 and 0.46, respectively), indicating that 50 mg/kg/biweekly injection of methotrexate was well tolerated. However, mean body weight for mice treated with gemcitabine alone decreased significantly by the end of the study (^**^p = 0.01), indicating that 80 mg/kg/biweekly injection of gemcitabine had an adverse cytotoxic effect on normal tissues. Importantly, mean body weight for mice treated with the combination of methotrexate and gemcitabine did not show a significant decrease (*p* = 0.38), suggesting that methotrexate protected normal proliferating cells in these mice from systemic gemcitabine toxicity.

## DISCUSSION

When patients receive a nucleoside analog drug such as gemcitabine, all proliferating cells, healthy and malignant, are affected. It is likely that cancer cells, because they proliferate more rapidly than normal host cells, incorporate higher levels of nucleoside analog, thus leading to greater cytotoxicity and a therapeutic advantage. This underlying principle of differential toxicity is a hallmark of systemic cancer treatment strategies. While chemotherapeutic protocols optimized toward differential toxicity can be effective, drug dosages are still constrained by deleterious side effects resulting from general cytotoxicity. We address this issue by leveraging several observations related to the functions and pathologies associated with CKS protein overexpression in order to selectively sensitize malignant cells with high CKS protein levels to nucleoside analog drugs, relative to normal host cells.

Cks1 and Cks2 have been shown to be overexpressed in many cancers and this phenotype correlates with poorer prognosis [11, 16, 18, 27]. The molecular mechanisms leading to CKS protein overexpression are not completely understood. At present, it is known that Cks1 overexpression can occur due to gene amplification in breast cancer [12] and myeloma [28, 29], and *CKS1* is a transcriptional target of c-Myc, B-RAF, and cyclin D1 [30, 31]. We previously reported that under replication stress, cells overexpressing CKS proteins bypass the replication stress checkpoint [18]. Since oncoproteins such as cyclin E [32] and c-Myc [33], when overexpressed, produce replication stress, CKS protein overexpression likely helps premalignant cells evade oncoprotein-induced replication stress that limits their expansion.

However, this advantage confers a potential vulnerability that could be exploited therapeutically. We hypothesize that while tumor cells overexpressing Cks1, Cks2, or both, can override the replication stress checkpoint, this renders them vulnerable to nucleoside analog drugs under conditions of replication stress, e.g., triggered by methotrexate. Conversely, non-malignant proliferating host cells that generally have low CKS protein levels would be rescued from the toxic effects of the nucleoside analog because checkpoint activation would halt DNA replication, thus preventing the incorporation of the analog. Our results support this hypothesis in two ways. First, cultured cells engineered to overexpress Cks1 or Cks2 were shown to be vulnerable to gemcitabine treatment in the presence of methotrexate, whereas parental control cells with low CKS protein expression were much less affected. Second, when a collection of triple negative breast cancer cell lines was stratified based on an estimate of aggregate *CKS1* and *CKS2* mRNA levels as surrogates for protein levels, there was a strong correlation between total CKS protein levels and sensitivity to gemcitabine in the presence of replication stress. Importantly, replication stress rescued a non-transformed mammary epithelial cell line (HME-1) from gemcitabine-induced toxicity to a greater degree than any of the breast cancer cell lines. Considering HME-1 cells as a surrogate for proliferating non-malignant tissue, this result suggests that high degrees of selective cytotoxicity can be achieved by treating CKS protein-overexpressing cancers with a combination of a replication stress inducer and a toxic nucleoside analog. This concept was investigated in a mouse xenograft model where MCF-7 human breast cancer cells engineered to overexpress Cks2 were compared to their controls (with low Cks2 levels) for tumor growth responses after implantation into mammary fat pads of female nude mice. At this orthotopic site, MCF-7 cells formed tumors regardless of CKS protein expression levels. Importantly, control tumors with low Cks2 expression were protected from gemcitabine-mediated toxicity by methotrexate. In contrast, Cks2-overexpressing tumors were not. This study employed a single dose level of gemcitabine to explore proof-of-principle evidence. The chosen gemcitabine dosage resulted in significant weight loss in tumor-bearing mice during the study, when gemcitabine was the only drug given, indicating non-specific toxicity. However, this toxicity was not seen in tumor-bearing animals receiving the methotrexate-gemcitabine combination, suggesting that replication stress could protect animals from chemotherapy-induced systemic toxicity. Therefore, with the protective effect of methotrexate, it is potentially possible to increase the dose of gemcitabine to levels that are highly effective against CKS protein-overexpressing tumors while sparing normal, proliferative tissues that generally express low levels of CKS proteins.

We have previously shown that CKS protein overexpression sensitizes cultured cells and xenograft tumors to a chemotherapeutic agent. MCF-7 cells and tumors programmed to overexpress Cks1 or Cks2 exhibited increased sensitivity to the drug 5-flourouracil (5-FU) [21]. In that study, we hypothesized that checkpoint override was responsible for sensitivity to 5-FU, although the mechanism was not probed in detail, as we have done here. In the context of the current study, it is interesting to note that 5-FU functions both as a replication stressor by inhibiting thymidylate synthase and as a nucleoside analog, by being converted to dFUTP and subsequently being incorporated into DNA. Therefore 5-FU alone can most likely produce conditions similar to the combinatorial approach utilized in the current study. However, the approach described in current study has the significant advantage of allowing optimization of the replication stress-inducing agent and the nucleoside analog separately, thereby providing greater selectivity.

CKS proteins are frequently overexpressed in a broad spectrum of human tumors. This implies that many cancer patients might benefit from a combinatorial therapeutic strategy that includes a replication stress inducer and a nucleoside analog. Importantly, successful estimation of CKS protein levels in tumor-derived cell lines using *CKS* mRNA levels suggests that qPCR-based analysis of *CKS* mRNA expression in tumor biopsy samples could be a reliable readout for selection of patient populations likely to respond and benefit from the proposed combinatorial therapeutic strategy.

It has been reported that combined administration of methotrexate and gemcitabine did not cause treatment-related mortality or limiting hematological toxicity among patients with malignant pleural mesothelioma [34]. Although there was no rationale involving CKS protein expression, this study indicates that the combination of methotrexate and gemcitabine is tolerated and feasible in a clinical context.

## MATERIALS AND METHODS

### Reagents

Primary Cks1/2 antibody was purchased from Santa Cruz Biotechnology, Inc. HRP-conjugated anti-rabbit secondary antibody was purchased from Jackson Laboratories. Drugs used included: gemcitabine hydrochloride (≥ 98%) (Sigma-Aldrich); methotrexate hydrate (≥ 98%) (Sigma-Aldrich); gemcitabine ^13^C^15^N_2_ hydrochloride (≥ 98.25%) (Clearsynth); mimosine (Indofine Chemical Co.); and thymidine (Fisher Scientific). Puromycin (Gemini Bio-Products) was used to select for CKS-overexpressing cells.

### Tissue culture

Newborn calf serum (NCS) was purchased from Gemini Bio-Products and tissue culture media were purchased from Thermo Fischer. BT549 cells were cultured in RPMI-1640 medium supplemented with 10% NCS and 1% PSQ (100 U/mL penicillin, 100 U/mL streptomycin, and 100 U/mL, and 2 mM _L_-glutamine; Thermo Fisher). HME-1 cells were cultured in MCDB 131 medium supplemented with 1% NCS, 1% PSQ, 70 μg/mL bovine pituitary extract (Hammond Cell Tech), 5 μg/mL human holo-transferrin (Sigma-Aldrich), 0.5 μg/mL hydrocortisone (Sigma-Aldrich), 10 ng/mL human epithelial growth factor, and 5 μg/mL insulin (Sigma-Aldrich). HeLa, HEK293A, and MDA-MB-231 cells were cultured in DMEM medium supplemented with 10% NCS and 1% PSQ. MCF-7 cells were cultured in RPMI-1640 medium supplemented with 10% NCS, 1% PSQ, 10 μg/mL insulin, and 10 nM β-estrodial estrogen (Sigma-Aldrich). MDA-MB-468 cells were cultured in DMEM/F-12 medium supplemented with 10% NCS and 1% PSQ. SUM149PT cells were cultured in Ham’s F-12 medium supplemented with 5% NCS, 1% PSQ, 5 μg/mL insulin, and 1 μg/mL hydrocortisone.

To generate HeLa and HEK293A transductants stably overexpressing Cks1 or Cks2, retroviral vectors expressing Cks1 or Cks2 with C-terminal FLAG-tags were first transfected by the calcium phosphate approach into Phoenix cells. Retroviral vectors were created based on the pQCXIP bicistronic retroviral plasmid (Addgene). Supernatants were collected 48 hrs post-transfection, filtered using a 0.45 μm polyethersulfone membrane, and added to HeLa or HEK293A cells that had been seeded for 24 hrs. After 48 hrs, transductants were selected in 5 μg/mL puromycin. Mixed populations were expanded and CKS expression levels were determined by qRT-PCR and western blotting.

For generation of stable Cks1/2-overexpressing MCF-7 cell populations, cells were transduced with lentiviruses that expressed Cks1 or Cks2 with C-terminal FLAG-tags. Lentiviral expression vectors were generated using vector pLenti CMV Puro DEST (Addgene). 48 hrs post-transduction, cells were selected in 5 μg/mL puromycin. Mixed populations were expanded and CKS levels were determined by qRT-PCR and western blotting.

All cell lines were authenticated either by the American Type Culture Collection or the Sanford Burnham Prebys Medical Discovery Institute Genomics Core Facility.

### Quantitative real time-PCR analysis

Cells were grown on 10-cm plates (Corning Inc.) to ∼90% confluency before harvesting. Total RNA was purified using the RNeasy Mini Kit (Qiagen). RNA (20 ng/μL) was reverse transcribed, amplified, and quantified using the BioRad CFX96 qRT-PCR detection system with PerfeCTa Sybr Green Supermix (Quanta BioSciences). *CKS1/2* expression levels were referenced to *CDK2* mRNA levels using the formula 2^CT(control mRNA)–CT(mRNA of interest)^ where CT is the threshold cycle. Primers were obtained from Integrated DNA Technology. Primers for *CDK2* were (forward) 5′GTTGAGGAACTGAGATGCGG 3′ and (reverse) 5′ TTGTTCTTGGATGTGGGGAG 3′. Primers for *CKS1* were (forward) 5′ CTGATGTCTGAATCTGAATGGAGG 3′ and (reverse) 5′TTTCTTTGGTTTCTTGGGTAGTGG 3′. Primers for *CKS2* were (forward) 5′GAAGAGGA GTGGAGGAGACTT 3′and (reverse) 5′ GTTGATCT TTTGGAAGAGGTCGT 3′.

### Clonogenic rescue assay

Cells were seeded in triplicate in six-well plates (Corning Inc) for 24-48 hrs before drug treatment for 48 hrs. Cells were then released in drug-free media and allowed to recover for 14-21 days. Colonies were stained with crystal violet (Sigma-Aldrich) and counted. Images of stained colonies were captured using the Odyssey Imaging System (Li-Cor).

### Cell death assessment

Cells were plated at 5 × 10^3^ cells per well for 24 hours and then treated with drugs for 48 hrs. Cells were then released for 48 hrs before being subjected to apoptotic assessment. For apoptosis detection, a homogeneous caspase-3/−7 fluorometric assay kit was used (Promega). Cells were permeabilized and incubated with pro-fluorescent rhodamine 110 for 1-2 hrs at room temperature in the dark. Presence of activated caspase-3/−7 cleaves the DEVD tetrapeptide on pro-fluorescent rhodamine 110, causing the molecule to fluoresce. Signal intensity was measured using a photometer at wavelengths 485 nm (excitation) and 530 nm (emission).

### Flow cytometry analysis

1 × 10^6^ cells were seeded in 10-cm plates for 24 hrs prior to drug treatment for 48 hrs. After harvesting, the cells were fixed overnight in 70:30 EtOH:PBS solution. After overnight fixation, cells were pelleted and permeabilized in 1 mL of 1% BSA/0.5% Tween-20/PBS. RNase solution was made by boiling 10 mg of RNase in 1 mL of 15 mM NaCl/50 nM tris solution for 15 minutes. 1 mL-RNase solution was combined with 9 mL of PBS-0.1% triton and 200 μL of 1 mg/mL propidium iodide (PI). After cells were pelleted by centrifugation, the pellets were resuspended in PI staining solution and incubated at room temperature for 2 hrs in the dark before flow cytometric analysis (ACEA Biosciences NovoCyte). Data analysis was conducted using FlowJo v.10.1 software.

### Heavy gemcitabine incorporation assay

1 × 10^6^ cells were seeded in 10-cm plates for 24 hrs before treatment with heavy gemcitabine with(out) other drugs for 48 hrs. Once harvested, the cells are washed and pelleted in 400 μL of 80:20 MeOH:H_2_O. Three cycles of where the resuspensions were snap-frozen in liquid N_2_ for 10 mins and then removed, thawed, and sonicated were carried out, after which the samples were vortexed for 1 min and then centrifuged at 4°C for 30 mins at 15,000 *g* to pellet insoluble material. After centrifugation, the supernatants were harvested and dried overnight. Purified DNA samples were reconstituted in 100 μL of ultra-pure H_2_O and subjected to mass spectrometric analysis.

### Protein quantification

Protein quantification via western blotting was done by first preparing extracts from pelleted cells lysed in RIPA buffer (Life Technologies) supplemented with 2x protease and phosphatase inhibitors (Roche Life Science). Proteins were resolved on 4–12% Bis-Tris gradient gels (Life Technologies) and transferred to nitrocellulose membranes using an iBlot Gel Transfer Device (Life Technologies). Amido black staining was used to calibrate loading. Membranes were blocked in TBST containing 5% w/v milk at room temperature for 1 hr and then incubated overnight in primary antibody (1:1,000) at 4°C. Membranes were then washed three times with TBST at 15-min intervals and then incubated with HPR-conjugated secondary antibody (1:2,500) for 1 hr at room temperature. Membranes were then washed again and developed using ECL technology.

### Animal experiments

Female nude (*nu/nu*) mice (Jackson Laboratories) were fed irradiated chow and water infused with trimethoprim/sulfamethoxazole (TMS) to minimize bacterial infection risk. At age 7 weeks, mice were implanted with a 17β-estradiol pellet (0.72 mg; Innovative Research of America), a dose generating systemic estrogen levels equivalent to that of human females mid-menstrual cycle [35], prior to inoculation with 1 × 10^6^ MCF-7 cells (stably overexpressing Cks2 or control) suspended in 30 μL of unsupplemented RPMI 1640 medium. The cells were injected into the fourth, right, inguinal mammary fat pad. Mice were randomly divided into four groups and biweekly intraperitoneal injection of 0.9% saline, methotrexate (50 mg/kg) [36], gemcitabine (80 mg/kg) [37], or the combination of methotrexate and gemcitabine began once tumor volume reached ∼50 mm^3^. Tumor volume was measured biweekly using digital calipers (Traceable) and calculated as (width^2^ × length)/2 = mm^3^ [21, 38]. Mice were weighed each time tumor volume was measured. Mice were euthanized 28 days after drug administration began, or if tumors size reached 1.5 cm in any direction, or if the tumor begins to ulcerate. Animal work complied with NIH and TSRI guidelines (TSRI is AAALAC accredited).

### Statistical analyses

Unless otherwise stated, statistical analyses were carried out using two-sided *t*-tests (to compare two groups), or one-way ANOVA (to compare three groups), and deemed significant if *p* < 0.05. Pooled data were expressed as mean (or normalized mean) ± SEM.

## SUPPLEMENTARY MATERIALS FIGURE



## Abbreviations

CDK: Cyclin-dependent kinase
Cks1/2: Cyclin-dependent kinase subunit 1/2
GEM: Gemcitabine
MTX: Methotrexate
MIM: Mimosine
PI: Propidium iodide
qRT-PCR: Quantitative reverse transcription polymerase chain reaction
THY: Thymidine
TNBC: Triple-negative breast cancer

## References

[1] Hayles J, Aves S, Nurse P (1986). suc1 is an essential gene involved in both the cell cycle and growth in fission yeast. Embo J.

[2] Hadwiger JA, Wittenberg C, Mendenhall MD, Reed SI (1989). The Saccharomyces cerevisiae CKS1 gene, a homolog of the Schizosaccharomyces pombe suc1+ gene, encodes a subunit of the Cdc28 protein kinase complex. Mol Cell Biol.

[3] Richardson HE, Stueland CS, Thomas J, Russell P, Reed SI (1990). Human cDNAs encoding homologs of the small p34Cdc28/Cdc2-associated protein of Saccharomyces cerevisiae and Schizosaccharomyces pombe. Genes Dev.

[4] Bourne Y, Watson MH, Hickey MJ, Holmes W, Rocque W, Reed SI, Tainer JA (1996). Crystal structure and mutational analysis of the human CDK2 kinase complex with cell cycle-regulatory protein CksHs1. Cell.

[5] McGrath DA, Balog ERM, Kõivomägi M, Lucena R, Mai MV, Hirschi A, Kellogg DR, Loog M, Rubin SM (2013). Cks confers specificity to phosphorylation-dependent CDK signaling pathways. Nat Struct Mol Biol.

[6] Martinsson-Ahlzen HS, Liberal V, Grunenfelder B, Chaves SR, Spruck CH, Reed SI (2008). Cyclin-dependent kinase-associated proteins Cks1 and Cks2 are essential during early embryogenesis and for cell cycle progression in somatic cells. Mol Cell Biol.

[7] Spruck C, Strohmaier H, Watson M, Smith AP, Ryan A, Krek TW, Reed SI (2001). A CDK-independent function of mammalian Cks1: targeting of SCF(Skp2) to the CDK inhibitor p27Kip1. Mol Cell.

[8] Spruck CH, de Miguel MP, Smith AP, Ryan A, Stein P, Schultz RM, Lincoln AJ, Donovan PJ, Reed SI (2003). Requirement of Cks2 for the first metaphase/anaphase transition of mammalian meiosis. Science.

[9] Bornstein G, Bloom J, Sitry-Shevah D, Nakayama K, Pagano M, Hershko A (2003). Role of the SCFSkp2 ubiquitin ligase in the degradation of p21Cip1 in S phase. J Biol Chem.

[10] Tedesco D, Lukas J, Reed SI (2002). The pRb-related protein p130 is regulated by phosphorylation-dependent proteolysis via the protein-ubiquitin ligase SCF(Skp2). Genes Dev.

[11] Su AI, Welsh JB, Sapinoso LM, Kern SG, Dimitrov P, Lapp H, Schultz PG, Powell SM, Moskaluk CA, Frierson HF (2001). Molecular classification of human carcinomas by use of gene expression signatures. Cancer Res.

[12] Wang XC, Tian J, Tian LL, Wu HL, Meng AM, Ma TH, Xiao J, Xiao XL, Li CH (2009). Role of Cks1 amplification and overexpression in breast cancer. Biochem Biophys Res Commun.

[13] Meihuali YM, Suguruhasegawa TS (2004). Genes associated with liver metastasis of colon cancer, identified by genome-wide cDNA microarray. International journal of oncology.

[14] Wong YF, Cheung TH, Tsao GS, Lo KW, Yim SF, Wang VW, Heung M, Chan S, Chan LK, Ho TW (2006). Genome-wide gene expression profiling of cervical cancer in Hong Kong women by oligonucleotide microarray. Int J Cancer.

[15] Lan Y, Zhang Y, Wang J, Lin C, Ittmann MM, Wang F (2008). Aberrant expression of Cks1 and Cks2 contributes to prostate tumorigenesis by promoting proliferation and inhibiting programmed cell death. International journal of cancer.

[16] Van't Veer LJ, Dai H, Van De Vijver MJ, He YD, Hart AA, Mao M, Peterse HL, van der Kooy K, Marton MJ, Witteveen AT (2002). Gene expression profiling predicts clinical outcome of breast cancer. Nature.

[17] Györffy B, Lanczky A, Eklund AC, Denkert C, Budczies J, Li Q, Szallasi Z (2010). An online survival analysis tool to rapidly assess the effect of 22,277 genes on breast cancer prognosis using microarray data of 1,809 patients. Breast cancer research and treatment.

[18] Liberal V, Martinsson-Ahlzen HS, Liberal J, Spruck CH, Widschwendter M, McGowan CH, Reed SI (2012). Cyclin-dependent kinase subunit (Cks) 1 or Cks2 overexpression overrides the DNA damage response barrier triggered by activated oncoproteins. Proc Natl Acad Sci U S A.

[19] Bartek J, Bartkova J, Lukas J (2007). DNA damage signalling guards against activated oncogenes and tumour progression. Oncogene.

[20] Bartkova J, Hořejší Z, Koed K, Krämer A, Tort F, Zieger K, Guldberg P, Sehested M, Nesland JM, Lukas C (2005). DNA damage response as a candidate anti-cancer barrier in early human tumorigenesis. Nature.

[21] del Rincon SV, Widschwendter M, Sun D, Ekholm-Reed S, Tat J, Teixeira LK, Ellederova Z, Grolieres E, Reed SI, Spruck C (2015). Cks overexpression enhances chemotherapeutic efficacy by overriding DNA damage checkpoints. Oncogene.

[22] Reid RJ, Du X, Sunjevaric I, Rayannavar V, Dittmar J, Bryant E, Maurer M, Rothstein R (2016). A Synthetic Dosage Lethal Genetic Interaction Between CKS1B and PLK1 Is Conserved in Yeast and Human Cancer Cells. Genetics.

[23] Herbert BS, Wright WE, Shay JW (2002). p16INK4a inactivation is not required to immortalize human mammary epithelial cells. Oncogene.

[24] Plunkett W, Huang P, Xu YZ, Heinemann V, Grunewald R, Gandhi V (1995). Gemcitabine: metabolism, mechanisms of action, and self-potentiation. Semin Oncol.

[25] Watson PA, Hanauske-Abel HH, Flint A, Lalande M (1991). Mimosine reversibly arrests cell cycle progression at the G1–S phase border. Cytometry.

[26] Wang G, Miskimins R, Miskimins WK (2000). Mimosine arrests cells in G1 by enhancing the levels of p27 Kip1. Exp Cell Res.

[27] Yu MH, Luo Y, Qin SL, Wang ZS, Mu YF, Zhong M (2015). Up-regulated CKS2 promotes tumor progression and predicts a poor prognosis in human colorectal cancer. Am J Cancer Res.

[28] Chang H, Qi X, Trieu Y, Xu W, Reader JC, Ning Y, Reece D (2006). Multiple myeloma patients with CKS1B gene amplification have a shorter progression-free survival post-autologous stem cell transplantation. Br J Haematol.

[29] Bahmanyar M, Qi X, Chang H (2013). Genomic aberrations in anaplastic multiple myeloma: high frequency of 1q21 (CKS1B) amplifications. Leukemia research.

[30] Keller UB, Old JB, Dorsey FC, Nilsson JA, Nilsson L, MacLean KH, Chung L, Yang C, Spruck C, Boyd K, Reed SI, Cleveland JL (2007). Myc targets Cks1 to provoke the suppression of p27Kip1, proliferation and lymphomagenesis. EMBO J.

[31] Bhatt K, Hu R, Spofford L, Aplin A (2007). Mutant B-RAF signaling and cyclin D1 regulate Cks1/S-phase kinase-associated protein 2-mediated degradation of p27Kip1 in human melanoma cells. Oncogene.

[32] Bartkova J, Rezaei N, Liontos M, Karakaidos P, Kletsas D, Issaeva N, Vassiliou LVF, Kolettas E, Niforou K, Zoumpourlis VC (2006). Oncogene-induced senescence is part of the tumorigenesis barrier imposed by DNA damage checkpoints. Nature.

[33] Dominguez-Sola D, Ying CY, Grandori C, Ruggiero L, Chen B, Li M, Galloway DA, Gu W, Gautier J, Dalla-Favera R (2007). Non-transcriptional control of DNA replication by c-Myc. Nature.

[34] Kuribayashi K, Miyata S, Fukuoka K, Murakami A, Yamada S, Tamura K, Hirayama N, Terada T, Tabata C, Fujimori Y (2013). Methotrexate and gemcitabine combination chemotherapy for the treatment of malignant pleural mesothelioma. Molecular and clinical oncology.

[35] Dall G, Vieusseux J, Unsworth A, Anderson R, Britt K (2015). Low dose, low cost estradiol pellets can support MCF-7 tumour growth in nude mice without bladder symptoms. Journal of Cancer.

[36] Cheng XL, Zhou TY, Li B, Li MY, Li L, Li ZQ, Lu W (2013). Methotrexate and 5-aminoimidazole-4-carboxamide riboside exert synergistic anticancer action against human breast cancer and hepatocellular carcinoma. Acta Pharmacologica Sinica.

[37] Qi H, Lu J, Li J, Wang M, Xu Y, Wang Y, Zhang H (2016). Enhanced Antitumor Activity of Monophosphate Ester Prodrugs of Gemcitabine: In Vitro and In Vivo Evaluation. Journal of Pharmaceutical Sciences.

[38] Tomayko MM, Reynolds CP (1989). Determination of subcutaneous tumor size in athymic (nude) mice. Cancer Chemotherapy and Pharmacology.

