# Supplementary material for: CKS protein overexpression renders tumors susceptible to a chemotherapeutic strategy that protects normal tissues
Source: Oncotarget. 2017 Dec 4;8(70):114911–23. doi: 10.18632/oncotarget.22931 (PMC5777741; doi:10.18632/oncotarget.22931)
Supplement: Supplementary file 1 [file oncotarget-08-114911-s001.pdf]

CKS protein overexpression renders tumors susceptible to a chemotherapeutic strategy that protects normal tissues

SUPPLEMENTARY MATERIALS

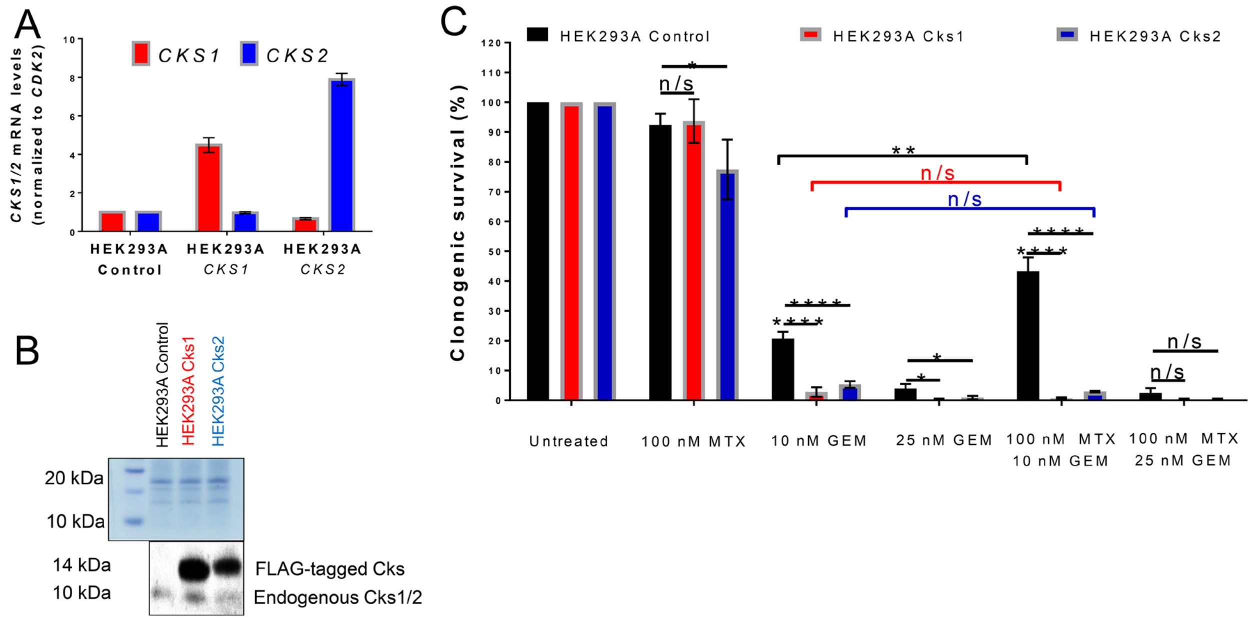

Supplementary Figure 1: CKS protein overexpression sensitizes, while low CKS protein expression protects proliferating HEK293A cells from gemcitabine induced toxicity under replication stress. HEK293A cells were transduced with retroviruses that overexpress Cks1 or Cks2 and expression was measured by qRT-PCR (**A**) and western blotting (**B**). *CKS* mRNA levels were normalized to *CDK2* mRNA. Amido black staining was used to calibrate protein loading. (**C**) Quantification of two ($N = 2$) separate clonal survival experiments performed in triplicate (two-sided t-test: $^{*}p < 0.05$, $^{**}p \leq 0.01$, and $^{****}p \leq 0.0001$). Data are presented as normalized mean ± SEM.
